# Accidental ingestion of multiple magnetic beads by children and their impact on the gastrointestinal tract: a single-center study

**DOI:** 10.1186/s12887-023-04425-z

**Published:** 2024-01-03

**Authors:** Xian-Ling Li, Qin-Ming Zhang, Shou-Yan Lu, Ting-Ting Liu, Shuan-Ling Li, Long Chen, Fang-Nan Xie, Li Wang, Chuang-Hui Zhang, Da-Yong Wang, Liu-Ming Huang

**Affiliations:** 1 Department of Surgery, Beijing Children’s Hospital, Capital Medical University, National Center for Children’s Health, 56 Nanlishi Road, Xicheng District, 100045 Beijing, China; 2 Department of Surgery, Beijing United Family Hospital, 100015 Beijing, China; 3 Department of Surgery, Beijing Aiyuhua Women’s and Children’s Hospital, 100176 Beijing, China

**Keywords:** Children, Foreign bodies in digestive tract, Magnetic beads

## Abstract

**Objective:**

In this study, we aimed to enhance the treatment protocols and help understand the harm caused by the accidental ingestion of magnetic beads by children.

**Methods:**

Data were collected from 72 children with multiple gastrointestinal perforations or gastrointestinal obstructions. The 72 pediatric patients were divided into a perforation and a non-perforation group. The data collected for the analysis included the gender, age, medical history, place of residence (rural or urban), and symptoms along with the educational background of the caregiver, the location and quantity of any foreign bodies discovered during the procedure, whether perforation was confirmed during the procedure, and the number of times magnetic beads had been accidentally ingested.

**Results:**

The accuracy rate of preoperative gastrointestinal perforation diagnosis via ultrasound was 71%, while that of the upright abdominal X-ray method was only 46%. In terms of symptoms, the risk of perforation was 13.844 and 12.703 times greater in pediatric patients who experienced vomiting and abdominal pain with vomiting and abdominal distension, respectively, compared to patients in an asymptomatic state. There were no statistical differences between the perforation and the non-perforation groups in terms of age, gender, medical history, and the number of magnetic beads ingested (*P* > 0.05); however, there were statistical differences in terms of white blood cell count (*P* = 0.048) and c-reactive protein levels (*P* = 0.033). A total of 56% of cases underwent a laparotomy along with perforation repair and 19% underwent gastroscopy along with laparotomy. All pediatric patients recovered without complications following surgery.

**Conclusion:**

Abdominal ultrasonography and/or upright abdominal X-ray analyses should be carried out as soon as possible in case of suspicion of accidental ingestion of magnetic beads by children. In most cases, immediate surgical intervention is required. Given the serious consequences of ingesting this type of foreign body, it is essential to inform parents and/or caregivers about the importance of preventing young children from using such products.

## Introduction

Cases of foreign bodies in the gastrointestinal tract are quite common in children’s emergency rooms. About 1.97% of the foreign bodies consumed are magnets. In general, between 80 and 90% of swallowed foreign bodies can spontaneously pass through the stomach; however, if they are large or pointy, they frequently need to be removed via an endoscopic procedure, with only around 1% of instances necessitating surgical intervention [1]. In most cases, if it is a single magnet, it will pass through the gut without causing any injury or problems, [2] however, if multiple magnets are inadvertently swallowed, they may group together in the gastrointestinal tract and compress the bowel wall, which can result in necrosis, perforation, intestinal blockage, fistula formation, intestinal volvulus, and even toxic shock.

Due to the sluggish and deceptive nature of the onset of symptoms in children who have ingested foreign bodies, diagnosis and treatment may be delayed, which could have disastrous and even life-threatening consequences [3–8]. Most children who consume multiple magnets require endoscopic treatment or surgery. Among those who undergo surgery, approximately 41% need to have a fistula or perforation repaired, and 22% need to have some degree of bowel resection [9].

In China, incidents of children ingesting multiple magnetic beads occasionally occur, with the mainstream media occasionally reporting on occurrences. There are still not enough detailed reports on this issue. In this paper, we report on 72 cases of gastrointestinal perforation or obstruction brought on by the accidental ingestion of multiple magnetic beads and discuss the optimal management strategy.

## Materials and methods

### General information

In this retrospective study, the clinical data of 136 children were treated for the presence of foreign bodies in the gastrointestinal tract at the Beijing Children’s Hospital (connected with Capital Medical University) from July 2007 to October 2021 were reviewed. The inclusion criterion was children who were treated for the presence of multiple magnetic foreign bodies in the gastrointestinal tract. Patients who ingested other types of gastrointestinal foreign bodies were excluded. Finally, a total of 72 (52.9%) patients with gastrointestinal perforation or obstruction by ingesting multiple magnetic beads were included. The primary outcomes of this study are symptoms on perforation. In addition to information on the symptoms, the perforation site or foreign body retention site, the number of foreign bodies, their location and number discovered during surgery, and whether perforation had been confirmed during the surgical procedure were collected as well as information related to the caregivers’ educational background, the residential type (rural or urban), the number of times magnetic beads had been accidentally swallowed, and other relevant data. All children underwent follow-up in an outpatient clinic at 3, 6, and 12 months after discharge.

### Clinical treatment methods

**Fig. 1:**
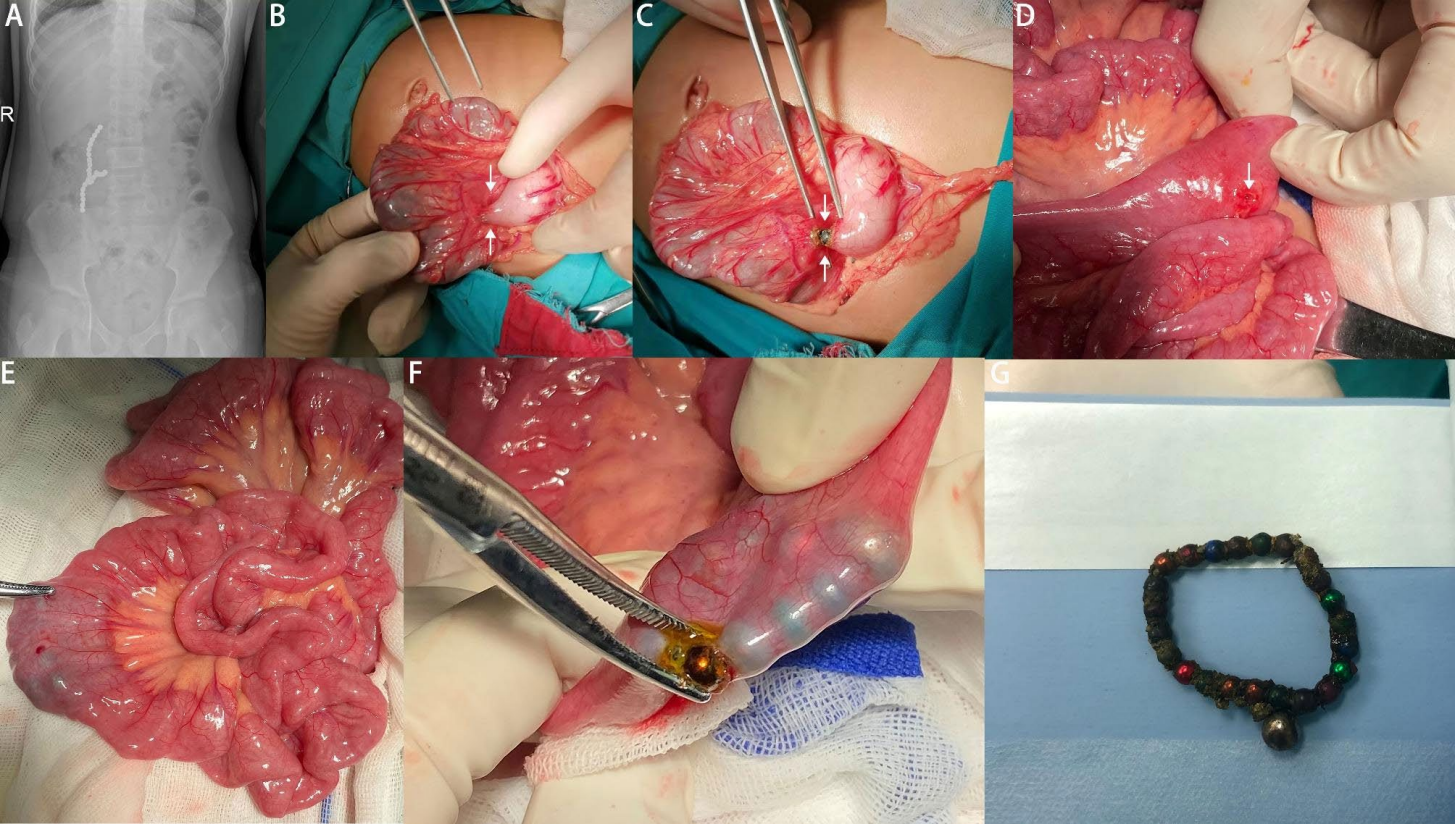
Diagnostic and surgical procedure. The surgical methods that were used for the removal of foreign bodies in the gastrointestinal tract. (**A**: abdominal upright X-ray; **B**: The two arrows point to an internal fistula; **C**: The two arrows point to a magnetic bead after incision of the fistula; **D**: The arrow points to a perforation of the intestine; **E**: The forceps is attracted by magnetic beads in the intestinal cavity; **F**: Magnetic beads after incision of intestinal wall; **G**: Magnetic beads after removal)

**Fig. 2:**
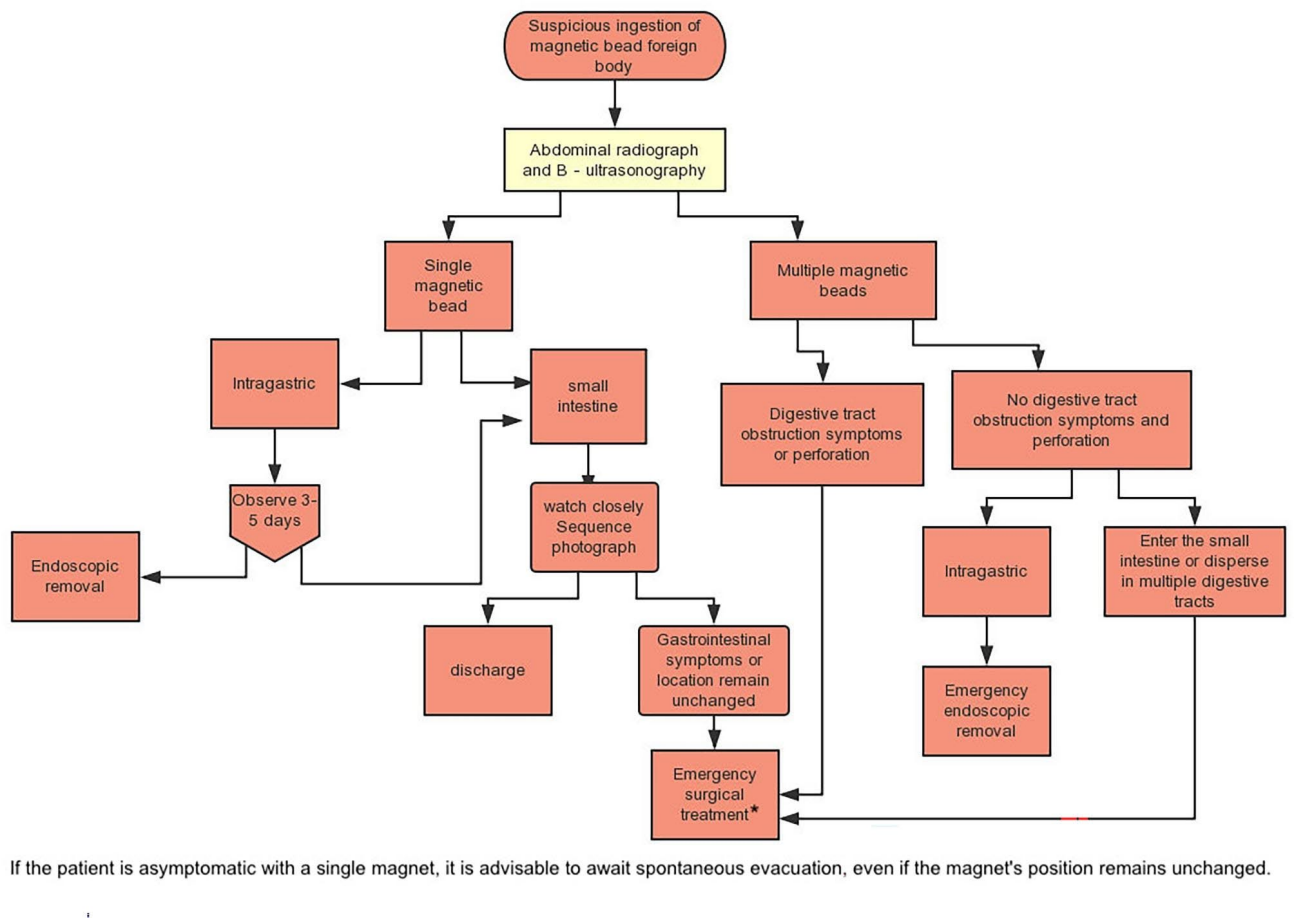
Beijing Children’s Hospital Affiliated with Capital Medical University - Diagnosis and Treatment Process of Accidental Ingestion of Magnetic Beads

Following the clinical diagnosis, the patients underwent active preoperative preparation, which included 6-hour pre-surgery fasting, gastrointestinal decompression, and situation-specific intravenous anti-inflammatory treatment. An exploratory laparotomy was considered if the magnetic bead was found in the duodenum, small intestine, or colon, if multiple beads were scattered in the stomach, or if there was a clear indication of gastrointestinal perforation. A gastroscopy was the preferred option if ultrasound and abdominal upright X-ray analyses revealed a magnetic bead in the stomach. The specific surgical treatment techniques are shown in Figs. 1 and 2. The third generation cephalosporins have been used for anti-inflammation.

### Statistical methods

Statistical analysis was performed using the IBM SPSS Statistics 21.0 software package, including descriptive statistical analysis of all counts and percentages. Chi-squared and Fisher’s exact tests were used for categorical variables, with frequencies presented as percentages. The Shapiro-Wilk test was used to assess normality for all numerical variables. If the groups’ data had a normal distribution, the mean ± standard deviation was used for statistical description, and a *t*-test was used to compare the groups. If a normal distribution was not observed, the median was used for statistical description, and a non-parametric test was used for comparison. Using a multivariate logistic regression method, all given *P* values were double-tailed. A difference of *P <* 0.05 was regarded as statistically significant.

## Result

Among the 72 patients, the age range was 0.6 to 15.6 years with an average age of 3.29 ± 3.33 years. The number of ingested magnetic beads varied from 2 to 40. In 49 cases, the ingestion of the magnetic beads occurred at home, whereas in the remaining 23 cases, they occurred in kindergarten or primary school. To confirm the diagnosis, standard abdominal upright X-ray and abdominal ultrasound analyses were conducted in the outpatient or emergency departments. After admission, a preoperative blood examination (white blood cell (WBC) count, C-reactive protein (CRP) test) was carried out as per protocol. All intestine and/or gastric perforations were identified during the surgical procedure. The 72 patients were divided into the perforation (n = 53; 33 males and 20 females) and the non-perforation (n = 19; 14 males and 5 females) groups, respectively. The medical history of the perforation group covered 8.1 ± 8.6 days while that of the non-perforation group covered 7.3 ± 7.7 days. The mean number of magnetic beads ingested in the perforation group was 11.3 ± 10.2 while that in the non-perforation group was 8.1 ± 7.5. The leukocyte/WBC count was 11.4 ± 3.1 (×10^9^) in the perforation group and 8.3 ± 2.2 (×10^9^) in the non-perforation group, while the CRP level was 28.0 ± 29.5 mg/l in the perforation group and 8.0 ± 0.0 mg/l in the non-perforation group.

There were 16 cases of abdominal pain, 8 cases of vomiting, 5 cases of fever, and 28 cases of abdominal pain combined with vomiting and abdominal distension; 15 patients were asymptomatic. The predominant symptoms were abdominal pain, vomiting, and abdominal distension, although an asymptomatic state was seen in 15 cases.

Patients who had symptoms of vomiting (2 cases) and abdominal pain with vomiting and abdominal distension (3 cases) had a 13.844- and 12.703-fold higher risk of perforation, respectively, compared to those who had no symptoms (risk value = 1) (see Table 1).

**Table 1:** The influence of different symptoms on perforation and non-perforation patients

Variables in the equation	B	S.E	Wals	df	Sig.	Exp (B)	EXP(B) 95% C.I.	
							lower limit	upper limit
symptom			9.949	4	0.041			
symptom(1)	1.654	0.859	3.708	1	0.054	5.225	0.971	28.123
symptom(2)	2.628	1.295	4.115	1	0.043	13.844	1.093	175.362
symptom(3)	2.542	0.881	8.324	1	0.004	12.703	2.260	71.421
symptom(4)	2.007	1.366	2.159	1	0.142	7.438	0.512	108.102
constant	-3.071	1.389	4.890	1	0.027	0.046		

Note: symptom(1), abdominal pain; symptom(2), vomiting; symptom(3), abdominal pain with vomiting and abdominal distension; symptom(4), fever

B, coefficient value; S.E., standard error; Wald, Chi-square value; df, degrees of freedom; Sig. P-value; Exp(B), odds ratio value

The accuracy rate of a preoperative B-scan ultrasound examination for gastrointestinal perforation was 71% (*P* = 0.005). In contrast, the accuracy of a preoperative abdominal radiograph examination with or without free gas under the diaphragm was only 46% (*P* = 0.539).

There were no statistically significant differences in terms of age, gender, medical history, and the number of magnetic beads ingested between the perforation and the non-perforation groups (*P* > 0.05), but there were statistically significant differences in terms of WBC count (*P* = 0.048) and CRP level (*P* = 0.033) between the two groups (Table 2).

**Table 2:** Comparison of General Conditions between Perforated and Non-perforated Patients

	Perforation group (n = 53)	Non-perforation group (n = 19)	P
Age	3.5 ± 3.4	3.2 ± 2.9	0.315
Gender			1.000
Male	33	14	
Female	20	5	
Medical history (days)	9.53 ± 17.49	11.87 ± 20.25	0.633
Number of magnetic beads	10.69 ± 10.50	7.05 ± 5.82	0.158
WBC count (×10^9^)	10.74 ± 4.14	8.74 ± 2.09	0.048
CRP levels (mg/L)	22.42 ± 29.16	7.78 ± 0.91	0.033

Note: WBC (white blood cell); CRP (C-reactive protein)

In terms of surgical treatment methods, the main methods performed were exploratory laparotomy (34%), intestinal perforation repair (19%), and laparoscopic exploration (23%). The other methods include gastroscopy (7%), intestinal adhesiplysis (6%), gastroscopic removal (5%), repair of gastric wall perforation (3%), and gastroscopy + laparotomy (3%). All patients recovered successfully and were discharged from the hospital with no complications reported during the follow-up to date.

A total of 246 magnetic-bead-type foreign bodies were found in the study group. There were 138 (56%) located in the jejunum and the ileum, which are sites prone to perforation, followed by 41 cases in colon (17%), 33 in duodenum (13%), 30 in stomach (12%), and 4 in esophagus (2%).

There was no significant difference in the number of accidentally ingested beads between the patients living in rural areas (46 cases) and those living in urban areas (26 cases) (*P* > 0.05).

There was a significant correlation between the number of incidences of ingesting magnetic beads and the educational level of the caregivers (primary school and below, middle school, and university) (*P* = 0.018). Specifically, the incidence rate was 14.062 times higher when the educational level involved primary school and below compared with the university level category.

## Discussion

Children are more likely to swallow multiple magnetic beads, which can cause catastrophic injuries like intestinal perforation, bowel strangulation, and necrosis [10, 11]. In the United States, it has been estimated that there are 100,000 cases of accidental foreign body ingestion per year, with more than 80% of the cases involving children under the age of five [12–15]. The incidence has been increasing over the past decade in various countries due to the widespread use of such magnets in products such as children’s toys [16–18]. This holds true for both children in cities and rural areas [3]. In this study, we found that the accuracy rate of preoperative gastrointestinal perforation diagnosis via ultrasound was higher than that of the upright abdominal X-ray method. The risk of perforation was greater in pediatric patients who experienced vomiting and abdominal pain with vomiting and abdominal distension, respectively, compared to patients in an asymptomatic state. The results of the present study suggest that there are no difference between the quantity and frequency of such accidental ingestion of magnetic beads in children living in rural and urban areas.

The magnetic balls noted above primarily comprise of neodymium–iron–boron (NdFeB) magnets, a type of artificial magnet with a very strong magnetic force that can attract weights up to 640 times that of their own weight [19]. Tsai et al. [20] found that NdFeB magnets attract one another when they are placed relatively near together (3.5–4.6 cm). Cox [21] discovered that once these magnets start to attract one another, the intestinal tissues may become sandwiched in the middle; the magnets would then be unable to separate on their own, which could result in rapid intestinal tract necrosis and perforation. The clinical symptoms may be minimal, and the imaging results may be atypical in some situations due to the wrapping of the omentum after perforation or the direct creation of an internal fistula [22]. In the present study, we found that the main symptoms were abdominal pain, vomiting, and abdominal distension followed by asymptomatic presentation; these findings were similar to those reported in existing studies [17, 23, 24]. Moreover, the risk of perforation due to symptoms vomiting and abdominal pain with vomiting and abdominal distension was higher than in cases involving asymptomatic presentation. Therefore, the risk of gastrointestinal perforation can be preliminarily determined based on the symptoms detected clinically.

To identify the number and location of the magnetic beads, parallel abdominal positive and lateral radiographs must be taken [3]. In the present study, the accuracy of diagnosing gastrointestinal perforation based on a preoperative B-scan ultrasound examination was higher compared to that based on the presence of free gas under the diaphragm. Many professional health organizations are aware of the risk of accidental magnet ingestion and advise seeking emergency medical attention in such circumstances [25]. There have been reports on various management approaches, but there is currently no agreement on the ideal management strategy [26]. The National Children’s Hospital Science Center and Beijing Children’s Hospital (affiliated with Capital Medical University) developed diagnosis and treatment processes for the ingestion of magnetic beads based on previously developed guidelines and algorithms and the magnet-type foreign body treatment process developed by the Shanghai Children’s Hospital [3, 20, 27, 28]. In numerous cases of multiple magnet ingestion, bowel resection, perforation and fistula repair have been conducted [10]. In the current study, the major surgical techniques used were laparotomy, intestinal perforation repair, and laparoscopic exploration. Compared with the non-surgical group, the peripheral WBC count and the CRP level were both higher in the surgical group. A high WBC count and/or CRP level suggest a high possibility of perforation, and surgical treatment is required as soon as possible in such cases [22]. There were no significant differences between the perforation and the non-perforation group in terms of mean age, gender, medical history, and the number of magnetic beads ingested. These findings were similar to those obtained by Zheng et al. [22] A year after this ban, the number of cases steadily increased, which may be explained by the unrecorded resale of such magnetic products [26]. Julie et al. reported that the severity of injuries caused by magnet consumption increased since 2009, with more instances requiring emergency surgery or hospitalization [29].

Supervision by parents and caregivers is a key factor in preventing injuries. Our study found a statistical correlation between the number of ingested magnetic beads and the educational level of children’s caregivers (primary school and below, middle school, and college/university). This revealed that caregivers with a higher level of education had a greater knowledge of the possible harm posed by magnetic beads. However, there was no difference between the number of rural and urban residents who accidentally ingested magnetic beads. Rosenfield reported that a re-examination of cases of multiple magnet ingestion in large pediatric hospitals revealed that the ingestion of multiple mini-magnets decreased significantly after a mandatory product recall, indicating that recalling such products could help reduce the number of injuries they cause [30]. The results of a survey undertaken by the American Academy of Pediatric Surgeons, as reported by Alicia, revealed that despite recent efforts to remove these products from the market, magnetic beads continue to pose a significant health risk to children, especially younger children [31]. The number of cases in 2018 and 2019 grew across all age groups, and cases involving the ingestion of magnets accounted for 39% of those reported since 2008 [25].

The limitations of this study include its reliance on data from a single center, which may limit the generalizability of the findings. Additionally, the relatively small sample size emphasizes the need for a larger dataset, which could be achieved through collaboration with multiple centers.

## Conclusion

Early symptoms following the accidental ingestion of multiple magnetic beads by children are often atypical, but the repercussions can be devastating. These magnets can gather in the gastrointestinal tract due through mutual attraction, causing life-threatening complications such as compression necrosis of the gastrointestinal wall, perforation, intestinal obstruction, and fistula formation. Abdominal B-scan ultrasound and abdominal standing X-ray analyses should be undertaken to confirm a diagnosis as soon as possible. Patients with higher WBC counts and/or CRP levels, as revealed by preoperative laboratory tests, should undergo active surgical treatment promptly. Concurrently, both society and the relevant authorities must control the production and sale of magnetic beads and related products, while parents and caregivers should be educated through educational school programs, social media, and public media channels about the unique risks posed by the ingestion of magnetic beads and how to prevent such instances from occurring.

## Data Availability

The datasets used and/or analysed during the current study available from the corresponding author on reasonable request.
